# Recent HIV infections and estimated HIV incidence among adolescents from key populations

**DOI:** 10.11606/s1518-8787.2024058005997

**Published:** 2024-10-11

**Authors:** Diana Zeballos, Fabiane Soares, Laio Magno, Celia Landmann Szwarcwald, Orlando Ferreira, Mateus Westin, Dirceu Greco, Alexandre Grangeiro, Inês Dourado

**Affiliations:** I Universidade Federal da Bahia. Instituto de Saúde Coletiva. Salvador, BA, Brasil Universidade Federal da Bahia Instituto de Saúde Coletiva Salvador BA Brasil; II Universidade do Estado da Bahia. Departamento de Ciências da Vida. Salvador, BA, Brasil Universidade do Estado da Bahia Departamento de Ciências da Vida Salvador BA Brasil; III Fundação Oswaldo Cruz. Instituto de Comunicação e Informação Científica e Tecnológica em Saúde. Rio de Janeiro, RJ, Brasil Fundação Oswaldo Cruz Instituto de Comunicação e Informação Científica e Tecnológica em Saúde Rio de Janeiro RJ Brasil; IV Universidade Federal do Rio de Janeiro. Instituto de Biologia. Laboratório de Virologia Molecular. Rio de Janeiro, RJ, Brasil Universidade Federal do Rio de Janeiro Instituto de Biologia Laboratório de Virologia Molecular Rio de Janeiro RJ Brasil; V Universidade Federal de Minas Gerais. Faculdade de Medicina. Belo Horizonte, MG, Brasil Universidade Federal de Minas Gerais Faculdade de Medicina Belo Horizonte MG Brasil; VI Universidade de São Paulo. Faculdade de Medicina. São Paulo, SP, Brasil Universidade de São Paulo Faculdade de Medicina São Paulo SP Brasil

**Keywords:** Adolescent, Sexual and Gender Minorities, HIV infections

## Abstract

**OBJECTIVE:**

To identify recent HIV-1 infection and estimate HIV incidence among adolescent men who have sex with men (AMSM) and transgender women (ATGW) in Brazil.

**METHODS:**

From January to December 2020, a cross-sectional analysis was conducted with baseline data from the PrEP1519 study, an HIV pre-exposure prophylaxis (PrEP) demonstration cohort in Brazil among sexually active AMSM/ATGW aged 15-19. For enrollment, participants were screened with a fourth-generation HIV rapid test. The recent infection testing algorithm (RITA) included a recency assay in blood specimens, viral load, and CD4 cell count prior to antiretroviral treatment use. Among these participants, RITA-based HIV incidence was estimated using a mean duration of recency infection of 214 days and a false-recent rate of 0.02.

**RESULTS:**

Out of the 494 participants screened, 21 tested positive for HIV. Following RITA, five adolescents had a recent HIV infection, 14 had long-term infections, and two did not have blood specimens available. We classified these two participants as long-term infection cases due to CD4 cell counts and previous use of antiretroviral treatment. Among those who tested positive, all but one were AMSM (94.7%), 73.6% were aged 18-19, and 76.2% were non-White. The HIV prevalence was 4.2%, and the estimated HIV incidence was 1.7%.

**CONCLUSIONS:**

The estimated incidence highlights the need for targeted HIV prevention interventions, such as PrEP, for sexual minority adolescents. Integrating RITA into routine HIV testing services for this population provides valuable information on the current HIV epidemic. This strategy can aid in monitoring the effectiveness of prevention efforts and improving early entry to HIV care.

## INTRODUCTION

Globally, the number of adolescents aged 10 to 19 years living with HIV reached approximately 1.7 million in 2022. This population accounts for approximately 4% of all individuals living with HIV and contributes to 10% of all new infections annually^1^. The HIV epidemic in Latin America is concentrated in key populations, with an estimated pooled prevalence among transgender women (TGW) of 19.9%^2^ and estimates ranging from 1.2 to 32.6% for men who have sex with men (MSM3). In Brazil, there has been a significant increase in Aids detection rates among young men over the past decade. Specifically, from 2011 to 2021, there was a 45.9% increase among men aged 15-19 years and a 26.2% increase among those aged 20-24 years^4^. Moreover, from 2000 and 2018, HIV incidence among young men in Brazil increased from 0.6 to 1.2 per 100 person years^5^.

Adolescence is a developmental period characterized by biological, behavioral, and social changes. During this period, adolescents gain independence and self- sufficiency at the same time that peer influence becomes relevant. Their organizational and planning skills are still developing, leading them to prioritize immediate needs and delay preventive behaviors^6^^), (^^7^. Actions that consider characteristics of this population are lacking regarding HIV prevention. Instead, a reduction in dialogue and guidance has been observed related to sexuality in schools^8^, as well as barriers to access to health services and testing for HIV due to requirements of parental consent^9^ and lack of services prepared to receive them^10^. Adolescents also have low rates of HIV testing and a considerable portion of them do not know their HIV status^11^. This translates into late HIV diagnosis and late entry into care and treatment^12^.

Given the challenges associated with estimating HIV incidence, which stem from the cost and complexity of longitudinal studies, recency assays have been developed to differentiate whether an individual’s HIV infection is recent or longstanding, being widely used as part of recent infection testing algorithms (RITAs) to estimate cross-sectional HIV incidence^13^. Moreover, the introduction of new effective strategies for HIV prevention such as pre- exposure prophylaxis for HIV (PrEP) hindered the evaluation of new prevention products that require non inferiority trials with larger sample sizes. In that scenario, estimating cross-sectional incidence has been proposed as a proxy to estimate counterfactual HIV incidence. In light of the implementation of new antiretroviral strategies for HIV prevention, such as PrEP and post-exposure prophylaxis (PEP), it is important to grasp the HIV incidence among adolescents from key populations. This understanding is crucial for tracking the transmission dynamics of the infection, evaluating the effectiveness of preventive measures, and ensuring timely access to HIV care. More recently, counterfactual HIV incidence has also been used to estimate as external control in prevention trials^14^. This study aims to estimate cross-sectional HIV incidence using a RITA among adolescents from key populations eligible for PrEP.

## METHODS

This is a cross-sectional study using baseline data from the PrEP1519 study, a daily oral PrEP demonstration cohort conducted in three Brazilian capital cities: Salvador, São Paulo, and Belo Horizonte. The study aims to evaluate the effectiveness of PrEP and other HIV combination prevention methods among adolescent MSM (AMSM) and adolescent TGW (ATGW). Adolescents self-identified as MSM or TGW, aged 15-19 years, who lived, studied, worked, or resided in one of the three cities, and who expressed the intention to use PrEP or other preventive method for HIV were eligible to participate in the study. Adolescents were reached via demand creation strategies that included online and in-person peer recruitment, as well as referrals from health services and non-governmental organizations^15^. Adolescents who agreed to participate in the study underwent screening with a fourth-generation rapid test (RT) that detects both HIV-1/2 antibodies and free HIV-1 p24 antigen. Those with a reactive result at baseline were enrolled in the HIV recency study. Participants who tested negative had the option to enroll in one of two arms: i) the PrEP arm, which included those who enrolled in daily oral PrEP with TDF/FTC in combination with other prevention methods (i.e., counseling, condoms, lubricants, and HIV self-test); ii) the non-PrEP arm, which included those who were eligible for PrEP but chose not to use drug prophylaxis and instead opted for other HIV combination prevention methods. More details of the PrEP1519 study have been published elsewhere^16^.

### Procedures

This study included all participants enrolled and screened for HIV infection in 2020. Participants with a reactive rapid test underwent a confirmatory RT and serological testing. In case of an invalid result, a fourth-generation rapid test was repeated. If the second test also returned an invalid result, two procedures were planned: return for testing after 14 days from their last sexual intercourse, if they were still within the window period; or, if 14 days had passed since their last intercourse, a fourth generation ELISA test was performed. Participants who presented symptoms suggestive of acute infection and had a negative result in the RT had blood collected for a fourth generation ELISA test and requested to return for testing after a 14-day period following their last intercourse. Adolescents diagnosed with HIV were referred and followed until the initiation of antiretroviral therapy (ART) in the health services of the Brazilian National Health System.

The recent infection testing algorithm (RITA) was used to classify adolescents into recent HIV infection or long-term infection, and included testing of blood specimens using Sedia LAg assay that detects antigen-driven antibody response (Sedia BioSciences, Portland, OR). To identify cases erroneously classified as recent infections by the Sedia LAg assay, quantification of viral load and T-CD4 cell count was conducted with <1,000 copies/mL and <350 cells/mm^3^ considered indicative of long-standing infection, respectively. Additionally, a search in national databases was performed to identify any previous use of ART for the treatment of HIV infection.

Besides HIV testing, specimens were collected for syphilis, hepatitis B and C, and HTLV I and II testing. A socio behavioral questionary was applied. AMSM and ATGW were the two subpopulations considered. Age was categorized into two groups (i.e., 15-17 and 18-19 years). Based on self-reported race/skin color categories, which included Black, Mixed-race, Asian, Indigenous, and White, a dichotomous variable was created, comparing White people with non-White people. Dichotomous variables (yes/no) were also created for condomless anal sex in the last six months, sex in exchange of money or favors (transactional sex), use of drugs and/or alcohol during sexual intercourse, situations of violence and discrimination related to the affective-sexual life, sexually transmitted infections in the last 12 months, PEP use in the last 12 months, and previous PrEP use.

### Statistical Analysis

Characteristics of participants by long-term infection and recent infection were expressed in terms of absolute and relative frequencies. Annualized HIV incidence rates were estimated using the updated UNAIDS/WHO “Using Recency Assays for HIV Surveillance 2022 Technical Guidance”^13^. RITA-based HIV incidence was obtained using a mean duration of recent infection of 171 days, which was estimated using an Odn <1.5 and a viral load above 1,000 copies/mL^17^; and a false-recent rate of 0.02. Analyses were performed using the R Studio software version 4.1.1

### Ethical Issues

The PrEP1519 study was conducted following the Brazilian (Resolution CNS no. 466, Brazil, 2012) and international research ethics guidelines. Research ethics committees of the World Health Organization, Universidade Federal da Bahia (UFBA) (#3,224,384), Universidade de São Paulo (#3,082,360), and Universidade Federal de Minas Gerais (#3,303,594) approved the conduction of the study. The participants aged 18 and 19 signed an informed consent form before participating. For those aged <18 years, each city followed a different protocol according to local court decisions: in Belo Horizonte the informed consent form had to be signed by the parents or guardian, followed by the assent form (AF) signed by the adolescents; in Salvador, there were two possibilities: i) the informed consent form was signed by a parent or guardian and the AF by the adolescent, or ii) only the AF was signed by the adolescent in cases in which the team’s psychologist and social worker judged that the family ties of the individual were broken or that the individual was at risk of physical, psychological, or moral violence due to the individual’s sexual orientation; and in São Paulo only the AF signed by the adolescents was required. All participants could withdraw consent at any stage of the process, as well as skip any questions that were perceived as being too sensitive, personal, or distressing. To guarantee confidentiality, all data were stored in a special and safe database, and no personally identifiable information was used for any public presentation or publication.

## RESULTS

**Figure 1:**
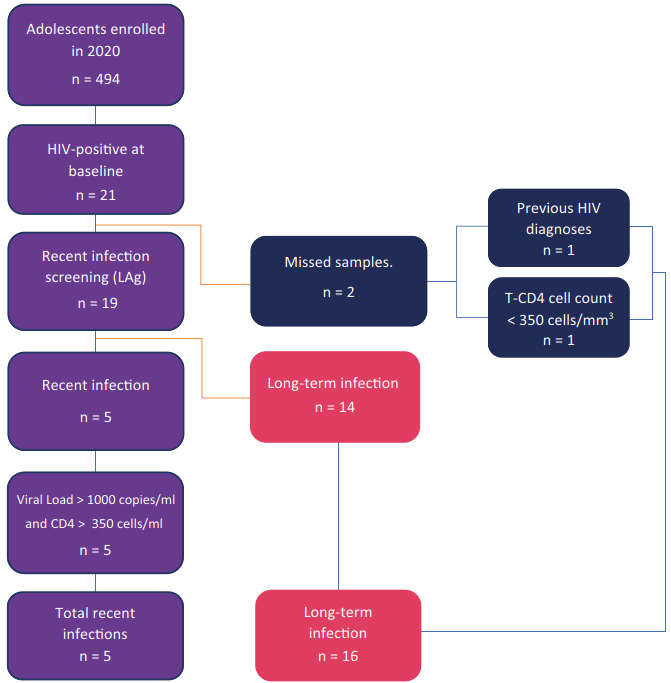
Recent infection testing algorithm of the PrEP1519 study.

A total of 494 adolescents were screened for HIV infection during 2020. Most of them were AMSM (93.7%), had 18-19 years (78.1%), and self-reported as non-Whites (73.3%) (Table 1). Out of the 494 participants screened, 473 (95.7%) had a negative RT result. Among the 21 adolescents who tested positive, 19 were evaluated using Lag avidity test and two samples were lost. In total, five adolescents (26.3%) were classified as recent HIV infection and 14 as long-term infection. Adolescents reporting previous use of PrEP or PEP were not individuals living with HIV. Consequently, none of these cases interfered with the RITA. We estimated HIV incidence for two scenarios, the first considering those two samples as missing information and the second considering them as long-standing infection, following our RITA, one due to a previous record in the Brazilian National CD4+/CD8+ T Lymphocyte Count and Viral Load Network Laboratory Test Control System (SISCEL) dated 12 months before the diagnosis in our study, and the second according to the T-CD4 cell count (Figure 1). The estimated HIV prevalence was 4.2% (95%CI: 2.78-6.44) and the HIV incidence based on RITA for the first scenario was 2.52% (95%CI: 0.17-4.87) and 2.26% (95%CI: 0.12-4.41) for the second scenario.

**Table 1:** Characteristics of adolescents tested for HIV in the PrEP1519 study in 2020.

Characteristic	Total
	n=494
	n (%)
Sub-population	
AMSM	463 (93.7)
ATGW	31 (6.3)
Age	
15-17 years old	108 (21.9)
18-19 years old	386 (78.1)
Race	
White	132 (26.7)
Non-White	362 (73.3)
Condomless anal sex in the last six months	
Yes	394 (79.8)
No	100 (20.2)
STIs in the previous 12 months	
Yes	109 (22.1)
No	385 (77.9)
PEP use in the last 12 months	
Yes	44 (8.9)
No	450 (91.1)
Frequent use of drugs and/or alcohol during sexual intercourse	
Yes	163 (33.0)
No	331 (67.0)
Transactional sexa	
Yes	71 (14.5)
No	417 (85.5)
Frequent situations of violence and discrimination related to the affective-sexual life	
Yes	159 (32.2)
No	335 (67.8)
Previous PrEP useb	
Yes	6 (1.3)
No	467 (98.7)

AMSM: adolescent men who have sex with men; ATGW: adolescent transgender women; STI: self-reported sexually transmitted infections; PEP: post-exposure prophylaxis; PrEP: pre-exposure prophylaxis.a n=488. b n=473

Table 2 compares participants’ characteristics by long-term and recent infection. Long-term infection was more frequent among those aged 18-19 years (81.2%) and the proportion of recent infections was higher among those aged 15-17 years (40.0%, p=0.57). Recent infection was more frequent among those with self-reported sexually transmitted infections (STIs) (33.3%) than in those without STIs (11.1%) in the previous 12 months (p=0.24).

**Table 2:** Comparison of the characteristics of adolescents by HIV infection status (long-term infection and recent infection).

Characteristic	Long-term infection	Recent infection	p value
	n=16	n=5	
Sub-population			0.57
AMSM	15 (75.0)	5 (25.0)	
ATGW	1 (100.0)	0 (0.0)	
Age			0.33
15-17 years old	3 (60.0)	2 (40.0)	
18-19 years old	13 (81.3)	3 (18.7)	
Race			0.82
White	4 (80.0)	1 (20.0)	
Non-white	12 (75.0)	4 (25.0)	
Condomless anal sex in the last six months			0.41
Yes	14 (73.7)	5 (26.3)	
No	2 (100.0)	0 (0.0)	
STI in the previous 12 months			0.24
Yes	8 (66.7)	4 (33.3)	
No	8 (88.9)	1 (11.1)	
PEP use in the last 12 months			-
Yes	0 (0.0)	0 (0.0)	
No	16 (76.2)	5 (23.8)	
Frequent use of drugs and/or alcohol during sexual intercourse			0.70
Yes	8 (80.0)	2 (20.0)	
No	8 (72.7)	3 (27.3)	
Transactional sex			0.95
Yes	3 (75.0)	1 (25.0)	
No	13 (76.5)	4 (23.5)	
Situations of violence and discrimination related to the affective-sexual life			0.72
Yes	5 (71.4)	2 (28.6)	
No	11 (78.6)	3 (21.4)	
Previous PrEP use			-
Yes	0 (0.0)	0 (0.0)	
No	16 (76.2)	5 (23.8)	

AMSM: adolescent men who have sex with men; ATG: adolescent transgender women; STI: self-reported sexually transmitted infections; PEP: post-exposure prophylaxis; PrEP: pre-exposure prophylaxis.

## DISCUSSION

HIV incidence was high among adolescent from key populations. These results highlight that sexual and gender minority adolescents are a major concern for HIV epidemic response efforts. Our results were almost four times higher than the incidence estimated via a RITA that used data from testing centers in Recife and Curitiba, in which individuals aged 13 years or older had incidence estimates of 0.05% (95%CI: 0.04-0.06) and 0.04 (95%CI: 0.03-0.05), respectively, and for young individuals 13 to 24 years old (0.06%) at both sites^18^. The HIV incidence estimated for males aged 15 to 24 years in 2018 for Brazil was 1.19% (95%CI: 1.09-1.645). In a study conducted in Rio de Janeiro from November 2004 to October 2005, participants from three voluntary counseling and testing centers were assessed using the BED-EIA HIV-1 incidence test. The study estimated an overall annualized HIV incidence of 1.68%. Notably, among MSM, the incidence was substantially higher at 11.96%^19^. Another study that enrolled MSM and TGW at a reference center in Rio de Janeiro found an annualized HIV incidence of 7.35% (95%CI: 5.76-9.2520). A more recent study conducted from January 2021 to May 2020 in Peru and Brazil including adult key populations estimated an annualized HIV incidence rate of 2.6%, in Brazil. This incidence was higher among the young population aged 18-24 (5.3%). However, they did not include adolescents aged 15-17 years^21^.

In the PrEP1519 cohort, over a 96-week period, the estimated HIV incidence was 1.6 per 100 adolescents who used PrEP. Notably, adolescents who seroconverted had levels of tenofovir diphosphate in dried blood spots below the lower limit of quantification, meaning low adherence to PrEP^22^. The incidence among adolescents prescribed PrEP was lower than HIV incidence at baseline, estimated following a RITA in our study, highlighting the effectiveness of PrEP and the relevance of estimating incidence using RITAs as reference to assess HIV prevention programs as new strategies are being implemented. Despite its effectiveness, daily oral PrEP may not fit into the routine of all adolescents, so there is a need to offer other choices of prevention methods, as well as the implementation of other PrEP modalities that may better suit the routine of adolescents, such as on-demand PrEP or injectable long-acting PrEP^23^. As these new methods are implemented, RITAs can help to estimate their impact on HIV incidence.

We observed that long-term infection was more frequent among older adolescents (81% *versus* 60%), suggesting that seroconversion may occur at younger ages but is detected later, and adolescents aged 15-17 have higher proportion of recent infections than those aged 18-19 (40% *versus* 18.7%). HIV testing and PrEP are crucial components of the combination HIV prevention strategy and previous studies revealed that access is limited among adolescents. In Brazil, a study involving 37,282 young male conscripts aged 17 to 22, found that only 5.1% of those who tested positive were aware of their HIV diagnosis^11^. Several studies have shown that younger ages stand out among the barriers to access to HIV testing among key populations^24^. In a qualitative study, adolescents mentioned parental consent as a barrier to access HIV testing services, as well as fear of stigma and discrimination often related to testing and the campaign itself, which emphasizes staying HIV negative rather than the benefits of knowing one’s HIV status^25^.

Additionally, condomless sex and previous STI diagnosis was high among adolescents that were screened to enter the PrEP1519 study. This underscores that adolescents are not adequately reached by sexual education programs and prevention services. On the one hand, this highlight a notable lack of discussion regarding these critical topics within families or schools. On the other hand, it indicates that the opportune moment to start prevention actions must precede this age group, approaching or even preceding the beginning of sexual intercourse. Without this, vulnerable adolescents will remain exposed to the risks of HIV infection and other STIs^8^.

The prevalence of HIV among adolescents from key populations was notably high, as highlighted in a previous study that examined factors associated with a specific sample from Salvador, one of the study sites. This prevalence reflects the high HIV burden among adolescents from sexual and gender minorities. When examined factors associated with HIV infection, including engaging in transactional sex, lower levels of schooling, and a lack of utilizing health services as a regular source of care, appeared as relevant^26^.

Some limitations need to be addressed. Firstly, the adolescents enrolled in the study were not randomly selected; instead, they were recruited via demand creation strategies specifically targeting individuals with profiles fitting to the main study. Consequently, these adolescents may represent a population at a higher risk of HIV, potentially suitable for PrEP use. Secondly, our analysis is based on 2020 data, a period likely influenced by the COVID-19 pandemic and social distancing measures. However, the PrEP1519 study adapted its approach during the pandemic by intensifying online demand creation strategies. The goal was to reach adolescents seeking socialization, engaging in sexual encounters, and participating in private sex parties during quarantine. This adaptation demonstrated that, despite the pandemic, the demand for PrEP and other HIV prevention services among adolescents from key populations persisted^27^.

In conclusion, the high recent HIV infection rates call attention to the need to prioritize adolescents from key populations for combined HIV prevention strategies. Integrating RITAs into routine HIV testing services for this population provides valuable information on the current HIV epidemic. Moreover, these statistics offer insight into the potential usefulness of this approach in evaluating the implementation of prevention strategies.
