# Erratum

**DOI:** 10.11606/s1518-8787.2025059005997err

**Published:** 2025-06-27

In the article “Recent HIV infections and estimated HIV incidence among adolescents from key populations”, DOI http://doi.org/10.11606/s1518-8787.2020054005997, published on the Revista de Saúde Pública. 2024;58: Suppl 1:3s, RSP corrects:

**How to cite (page 1):**

Where it reads:

http://doi.org/10.11606/s1518-8787.2020054005997

It should read:

http://doi.org/10.11606/s1518-8787.2024058005997

**Footnote (pages 1 to 9):**

Where it reads:

http://doi.org/10.11606/s1518-8787.2020054005997

It should read:

http://doi.org/10.11606/s1518-8787.2024058005997

